# Autoimmune susceptibility imposed by public TCRβ chains

**DOI:** 10.1038/srep37543

**Published:** 2016-11-21

**Authors:** Yunqian Zhao, Phuong Nguyen, Peter Vogel, Bofeng Li, Lindsay L. Jones, Terrence L. Geiger

**Affiliations:** 1 Department of Pathology, St. Jude Children’s Research Hospital, Memphis, TN, 38105, USA.

## Abstract

Although the TCR repertoire is highly diverse, a small fraction of TCR chains, referred to as public, preferentially form and are shared by most individuals. Prior studies indicated that public TCRβ may be preferentially deployed in autoimmunity. We hypothesized that if these TCRβ modulate the likelihood of a TCRαβ heterodimer productively engaging autoantigen, because they are widely present in the population and often high frequency within individual repertoires, they could also broadly influence repertoire responsiveness to specific autoantigens. We assess this here using a series of public and private TCRβ derived from autoimmune encephalomyelitis-associated TCR. Transgenic expression of public, but not private, disease-associated TCRβ paired with endogenously rearranged TCRα endowed unprimed T cells with autoantigen reactivity. Further, two of six public, but none of five private TCRβ provoked spontaneous early-onset autoimmunity in mice. Our findings indicate that single TCRβ are sufficient to confer on TCRαβ chains reactivity toward disease-associated autoantigens in the context of diverse TCRα. They further suggest that public TCR can skew autoimmune susceptibility, and that subsets of public TCR sequences may serve as disease- specific biomarkers or therapeutic targets.

T cell immunity is dependent upon T cell receptor (TCR) recognition of large numbers of antigenic peptides presented in the context of few major histocompatibility complex (MHC) proteins. The MHC is polymorphic and comprises the strongest genetic risk factor for autoimmune and other T cell-mediated diseases^1^,^2^,^3^. Only a small portion of the TCR repertoire recognizes any individual peptide-MHC complex, and these TCR complement disease-associated MHC to impose autoimmune risk. Somatic recombination and subsequent pairing of distinct TCR α and β chains endow T cells with a potential diversity of 10^15^–10^18^ unique TCRαβ heterodimers^4^,^5^. The number of circulating T cells is several orders of magnitude lower, and thus the TCR repertoire deployed in response to a particular disease-associated antigen should in theory be largely unique between individuals.

Despite their theoretical diversity, a small fraction of TCR α and β monomers preferentially form due to recombinatorial biases in early T cell development and are shared by most individuals^6^,^7^,^8^. These TCR, termed public, have been associated with a variety of immune responses, including autoimmunity^9^,^10^,^11^. Public TCR α or β chains pair with independently rearranged and largely unshared β or α chains to form unique TCRαβ heterodimers. Given that the ligand binding surface of TCR α and β contribute roughly equally to peptide-MHC recognition^12^, public TCRs, in which only a single TCR chain is shared, would not be expected to bias TCR recognition. However, it has also been shown that certain TRAV and TRBV chains are preferentially employed in specific immune responses. For instance, TRBV-1 is commonly found in synovial T cells in patients with reactive arthritis, and a Vβ13.1-derived CDR3 sequence was frequently seen in myelin basic protein reactive T cells clones from patients with multiple sclerosis (MS)^13^,^14^. In one case, a binding “hotspot” between a single TRBV and antigen-MHC ligand was identified, suggesting how a single TCR chain can bias recognition^9^. Considering this, public α or β chains which are fixed for V, J, and CDR3 sequences, may be capable of modulating the likelihood of a productive engagement between a TCRαβ heterodimer and autoantigen. In support of this hypothesis, one group reported that a substantial fraction of TCRαβ^+^ CD8 T cells from female mice transgenic for an H-Y specific TCRβ also recognized the H-Y antigen^15^.

Because public TCR chains are shared throughout a population and, due to their preferential formation, often present at high frequency^16^, if they do predispose TCR for recognition of specific autoantigens they may more broadly influence autoimmune susceptibility in individuals bearing risk-associated MHC. Our previous results using high-throughput sequencing of the TCRβ repertoire during myelin oligodendrocyte glycoprotein (MOG)-induced experimental autoimmune encephalomyelitis (EAE) indicated that a diverse public TCRβ repertoire is preferentially deployed relative to the non-shared, or private, pre-immune repertoire^17^. Here we assess the contribution of individual public and private TCRβ sequences to the autoimmune response during MOG_35-55_ -induced EAE. We describe mice that transgenically express 15 public or private disease-associated TCRβ, each of which pairs with endogenously rearranged TCRα. Public but not private TCRβ selectively imposed autoimmune risk, fostering autoantigen reactivity and even the development of spontaneous fulminant autoimmunity. Our findings demonstrate that single TCR chains can broadly influence repertoire reactivity and support the hypothesis that recognition biases imposed by public TCR contribute to autoimmune responses.

## Results

### Spontaneous autoimmunity mediated by a public TCRβ

To understand the composition and dynamics of autoimmune effector and regulatory repertoires, we previously performed saturation sequencing of splenic and CNS T cells from 12 mice with MOG_35-55_ -induced EAE and 5 healthy controls, analyzing >18 × 10^6^ CD4^+^Foxp3^–^ (Tconv) and Foxp3^+^ (Treg) TRBV13-2^+^ TCRβ^6^,^17^,^18^,^19^,^20^,^21^. TRBV13-2 is the dominant TCRβ in MOG-specific T cells^22^. Our results indicated the presence of a diverse, public TCR repertoire within the autoimmune response, and that T cells bearing public TCR were preferentially deployed relative to private TCR from the pre-immune repertoire^17^. This suggested a role for public sequences in predisposing the repertoire toward autoreactivity. To better define the impact of public TCRβ, we generated retroviral transgenic (retrogenic) mice on a TCRβ^−/−^ background that enforced the expression of public and private TCRβ sequences identified through these sequencing analyses and from MOG_35-55_ specific T cell hybridomas (Table 1). Mice retrogenic for the ovalbumin-specific OTIIβ chain were generated as an autoantigen non-specific control.

**Figure 1:**
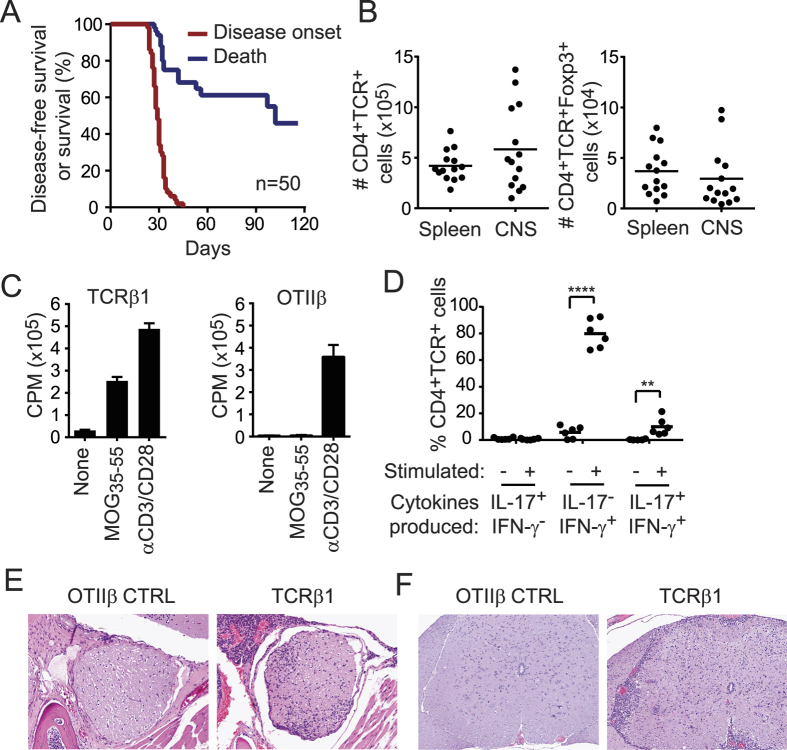
Enforced public TCRβ expression leads to spontaneous autoimmune encephalomyelitis. TCRβ^−/−^ Foxp3-GFP HPCs were transduced with TCRβ1 to generate retrogenic mice. (**A**) Kaplan Meier analysis of overall and disease-free survival. (**B**) Absolute number of CD4^+^TCR^+^ and CD4^+^TCR^+^Foxp3-GFP^+^ T cells in spleen and CNS of TCRβ1 mice with EAE. (**C**) Proliferation of splenic T cells from TCRβ1 or control retrogenic mice expressing the OTII TCRβ chain in response to MOG_35-55_ or mitogen measured by ^3^H-thymidine incorporation. (**D**) Percent of CNS- infiltrating TCRβ1T cells expressing IL-17, IFN-γ, or both IL-17 and IFN-γ in the absence or presence of *ex vivo* restimulation as determined by intracellular cytokine staining. (**E**,**F**) Histologic analyses of the CNS of TCRβ1 retrogenic mice showing a mixed infiltrate of lymphocytes, macrophages, and granulocytes, gliosis and perivascular cuffing in the septum, meninges, and optic nerve (**E**) and white tracts of the lumbar spinal cord (**F**) on day 28 of TCRβ1 but not OTIIβ control retrogenic mice. **p ≤ 0.01; ****p ≤ 0.0001.

TCRβ1 was found in splenic and CNS Treg and Tconv of all mice with EAE that were studied (Table 1)^17^. Impressively, TCRβ1 retrogenic mice uniformly developed spontaneous EAE at 4 weeks, corresponding to very early T cell engraftment (Fig. 1A). Indeed, numbers of T cells infiltrating the CNS at this early time were similar to numbers in the spleen (Fig. 1B). Mortality was >50% (Fig. 1A). CD4^+^Foxp3^−^, CD4^+^Foxp3^+^, and CD8^+^ T cells engrafted, and the CD69 activation marker was elevated in splenic and CNS T cells from diseased TCRβ1 mice relative to OTIIβ retrogenic mice (Supplementary Fig. S1). TCRβ1^+^ T cells proliferated vigorously in response to MOG_35-55_ (Fig. 1C). Splenic and CNS cells from TCRβ1 retrogenic mice also demonstrated Th1 and Th17 subset differentiation, which is associated with pathogenicity in EAE (Fig. 1D and Supplementary Fig. S1). Histologic analyses of the CNS of TCRβ1 retrogenic mice showed a mixed infiltrate of lymphocytes, macrophages, and granulocytes, gliosis and perivascular cuffing in the septum, meninges, optic nerve, and white tracts of the lumbar spinal cord, consistent with optico-spinal encephalomyelitis (Fig. 1E and F, Supplementary Fig. S1). Notably, disease in TCRβ1 retrogenic mice was markedly accelerated, increased in incidence, and more severe than our prior results with retrogenic mice expressing five different disease-associated private MOG- specific TCRαβ heterodimers^23^.

### TCRβ1 imposes MOG specificity on TCRαβ heterodimers

**Figure 2:**
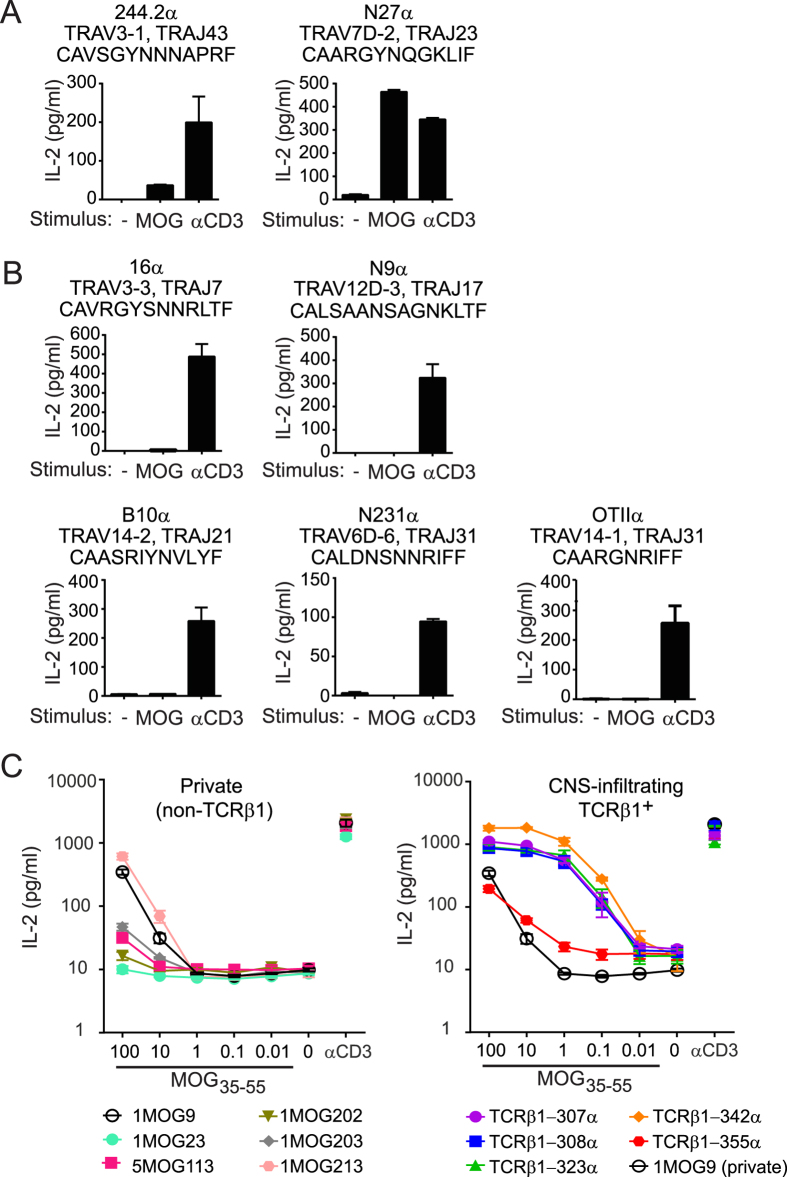
High sensitivity of TCRβ1^+^ TCR for MOG_35-55_. Seven TCRα chains derived from non-TCRβ1 TCR were cloned into the MSCV-I-GFP retroviral vector with TCRβ1. CD4^+^ 4G4 TCRαβ^−^ hybridoma cells were transduced and TCRαβ^+^ cells were sorted and stimulated with 100 μg/ml MOG_35-55_ or anti-CD3. IL-2 production was measured by ELISA at 24 h. (**A**) TCRα chains that conferred MOG_35-55_ responsiveness when paired with TCRβ1 and (**B**) TCRα chains that did not confer MOG_35-55_ responsiveness. Graphs are labeled with the TCRα name (top), TRAV and TRAJ segment use (middle), and the CDR3α sequence (bottom). (**C**) Five TCRα chains isolated from CNS-infiltrating TCRβ1^+^ TCR were linked to TCRβ1 in retroviral constructs as above (TCRβ1-342α, TCRβ1-307α, TCRβ1-308α, TCRβ1-323α, and TCRβ1-355α). Alternatively, retroviral constructs incorporating six private MOG_35-55_ -reactive TCRαβ (1MOG213, 1MOG203, 1MOG202, 5MOG113, 1MOG9, and 1MOG23) were similarly generated. Constructs were transduced into CD4^+^ 4G4 TCRαβ^−^ hybridoma cells and sorted for GFP-positivity and similar levels of TCR. Cell lines were stimulated with the indicated concentration of MOG_35-55_ or anti-CD3, and IL-2 production was measured at 24 h by ELISA. Private (non-TCRβ1) TCR are shown in the left panel and CNS-infiltrating TCRβ1^+^ TCR are shown in the right panel. Private TCR 1MOG9 is included in both panels to illustrate enhanced sensitivity of TCRβ1^+^ TCR relative to those from private MOG-reactive TCR. Data are representative of at least 3 independent experiments performed in duplicate.

We hypothesized that TCRβ1 supports MOG_35-55_ recognition by TCR with diverse TCRα. To establish pairing requirements, we first co-expressed TCRβ1 in CD4^+^ TCRαβ^−^ 4G4 hybridomas together with 7 TCRα chains that were isolated from non-TCRβ1 TCR. All α-TCRβ1 combinations were expressed, and two of the seven hybrid TCR responded to MOG_35-55,_ indicating that TCRβ1 can drive MOG_35-55_ responsiveness (Fig. 2A and B). TCRα form through recombination of the endogenous locus in developing thymocytes in TCRβ1 mice, and would thus be anticipated to be highly diverse. To assess the diversity of TCRα associated with TCRβ1 during the autoimmune response, we isolated TCRα cDNA from CNS- infiltrating T cells from 3 TCRβ1 mice by 5′ RACE. These were heterogeneous and did not overlap between mice (Supplemental Table S1), indicating that TCRβ1 is associated with diverse TCRα.

To quantify the functional responsiveness of TCRβ1^+^ TCR compared with private TCR, five TCRα derived from CNS-infiltrating TCRβ1^+^ T cells were cloned together with TCRβ1 into polycistronic retroviral constructs. In addition, six TCRαβ from private MOG_35-55_ -specific T cell hybriodomas were similarly cloned. These included previously described clones 1MOG9 and 5MOG113^23^. All TCR constructs were transduced into 4G4 CD4^+^ TCRαβ^−^ T cell hybridomas. Cells were sorted for co-expressed GFP and similar TCR levels. TCR avidity and MOG-sensitivity was functionally determined by stimulation with titrations of MOG_35-55,_ using IL-2 production as a readout. Four of the five TCRβ1-derived TCRs demonstrated a >2–3 log10 increased sensitivity for antigen and a dramatically increased maximal IL-2 response relative to any of the private TCR (Fig. 2C). This indicates that TCRβ1 imposes on TCRαβ an unusually high degree of responsiveness to MOG_35-55_ autoantigen.

**Figure 3:**
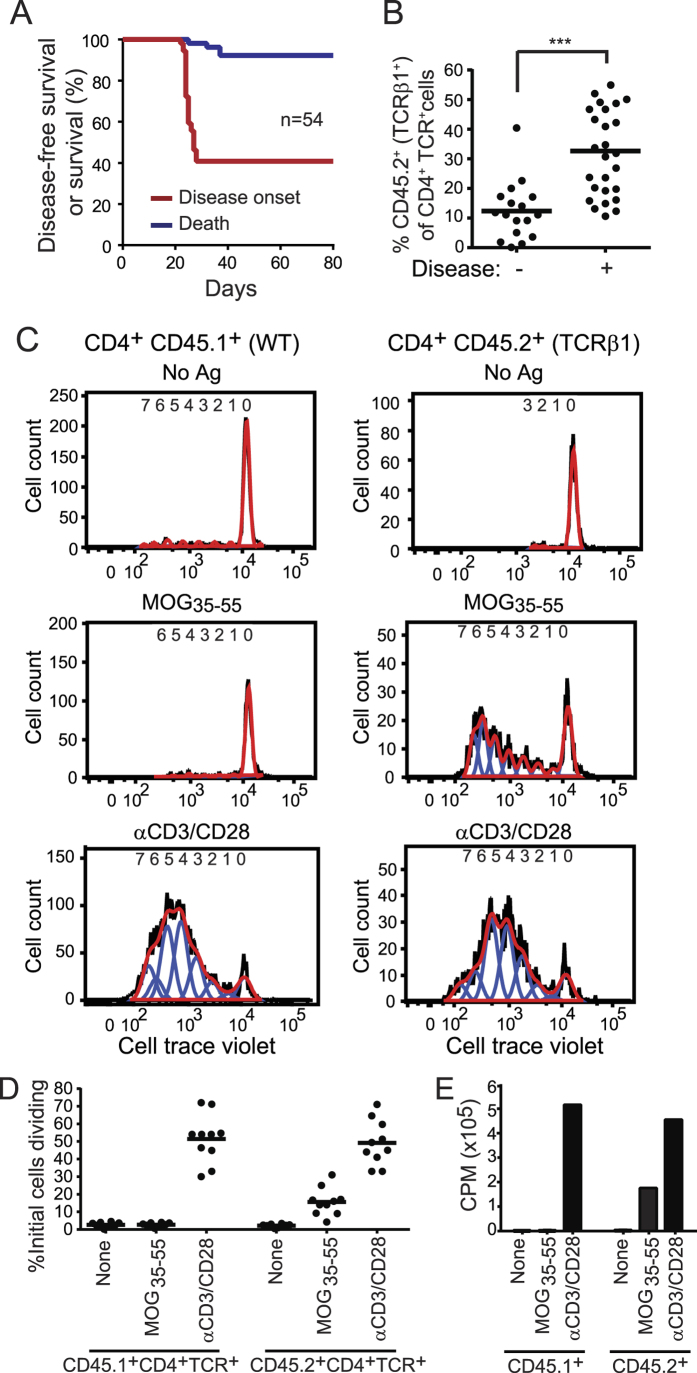
Unprimed T cells from disease-free TCRβ1 chimeric mice exhibit MOG responsiveness. (**A**) Disease-free and overall survival of mice chimeric for WT (CD45.1^+^) and TCRβ1 (CD45.2^+^) T cells are plotted. (**B**) Peripheral blood samples were collected at day 28. The percent of CD4^+^TCR^+^CD45.2^+^ (TCRβ1^+^) T cells among total CD4^+^TCR^+^ T cells in chimeric mice developing or not developing EAE is plotted. (**C**) *Ex vivo* proliferation of CD4^+^CD45.1^+^ (WT) and CD4^+^CD45.2^+^ (TCRβ1^+^) T cells from a representative 8 wk disease-free retrogenic mouse was measured by CellTrace Violet dilution 72 h after stimulation. Plots show total cell counts (black line), cell count data fit to proliferation model (red line), and individual proliferative generations (blue lines). (**D**) Summary data from T cells from individual mice stimulated as in (**C**). The magnitude of each division peak was divided by 2^n^, where n = division peak number, to estimate numbers of parental cells whose progeny populated an individual peak. (**E**) CD4^+^TCR^+^CD45.2^+^ (TCRβ1^+^) and CD4^+^TCR^+^CD45.1^+^ (WT) T cells were sorted from 8 wk chimeric mice without current or historical signs of EAE. The cells were stimulated as indicated and proliferation measured on day 3 by ^3^H-thymidine incorporation. ***p ≤ 0.001.

To determine whether non-transgenic T cells impede spontaneous EAE mediated by TCRβ1, we generated chimeric retrogenic mice. We mixed wild type (WT) CD45.1^+^CD45.2^−^ and smaller numbers of congenic TCRβ1-transduced CD45.1^−^CD45.2^+^ hematopoietic progenitor cells (HPCs). Approximately 40% of mice were protected from spontaneous disease. When EAE developed, symptoms were milder and mortality diminished, consistent with a protective role for the co-engrafted WT cells (Figs 1A and 3A). The ratio of TCRβ1 and WT T cells was measured in the peripheral blood with early engraftment (d28). TCRβ1^+^ cells were most often a minority, and significantly less frequent in mice that did not develop EAE compared with those that did (Fig. 3B). We anticipated that TCRβ1 would impose MOG-recognition on unprimed T cells in healthy animals. To test this, we analyzed MOG_35-55_ responsiveness in unprimed disease-free chimeric mice. T cells were labeled with cell trace violet, and stimulated either with MOG_35-55_ or αCD3/αCD28. TCRβ1^+^ CD45.2^+^ but not WT CD45.1^+^ T cells from unprimed disease-free mice proliferated strongly to MOG_35-55_ (Fig. 3C and D). An estimated 15.6 ± 7.8% of the initial population of CD45.2^+^ T cells responded to MOG_35-55_ compared to 49.2 ± 12.3% to control αCD3/CD28. Alternative analyses measuring ^3^H-thymidine incorporation in sorted and stimulated CD45.1^+^ and CD45.2^+^ T cells yielded similar results (Fig. 3E).

### Public but not private TCRβ confer myelin specificity and provoke spontaneous autoimmunity

TCRβ1 is to our knowledge the first example of a single TCR chain endowing a heterogeneous population of T cells with overt spontaneous autoreactivity in mice not otherwise susceptible to spontaneous autoimmunity. To more comprehensively define the impact of public TCRβ, we generated 14 additional TCRβ retrogenic mice (Table 1). Like TCRβ1, TCRβ2 − 6 were identified in ≥9 of 12 CNS’ and all spleens of mice with EAE that were analyzed (group 1; CNS-shared, public)^17^. TCRβ7-10 were seen in a single CNS at high frequency and shared in splenocytes to varying extents (group 2; CNS non-shared, public). TCRβ11-15 were wholly private (group 3; private). As previously reported, a large fraction of, though not all, CNS-infiltrating T cells in MOG-EAE recognize the MOG_35-55_ epitope^17^,^24^. To minimize the possibility that TCR selected for analysis were derived from non-specific bystander T cells, group 2 and 3 TCRβ were derived from high frequency CNS-infiltrating clones (β7-11) or from TCRαβ sequences isolated from private T cell clones demonstrated to recognize MOG_35-55_ autoantigen (β12-15). For each TCRβ, retrogenic mice were monitored for clinical disease for ^≥^120 days or until the development of disease, at which time all major organs were histologically assessed. T cells from additional disease-free mice were assayed for MOG_35-55_ -specific responsiveness.

**Figure 4:**
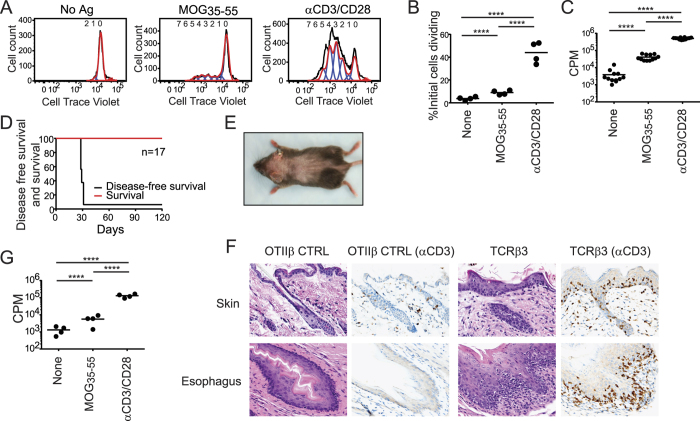
Heightened public TCRβ autoreactivity. (**A**) Proliferation of T cells from unprimed and disease-free retrogenic TCRβ4 mice was measured by dye dilution 72 h after stimulation as indicated. (**B**) The percent of initial TCRβ4 cells dividing in response to the indicated stimulus was calculated by dividing each division peak by 2^n^, where n = division number, to estimate initial cell numbers forming each peak. (**C**) Proliferative response of purified TCRβ4 T cells measured using ^3^H-thymidine incorporation. Circles indicate means of triplicates from individual mice. (**D**) Kaplan Meier analysis of alopecia-free and overall survival in TCRβ3 retrogenic mice. (**E**) The dorsal surface of a representative TCRβ3 mouse demonstrates extensive alopecia areata. (**F**) Premature catagen and inflammatory infiltrates in the follicular and interfollicular epidermis, and inflammatory infiltrates associated with diffuse thickening and hypercellularity of the squamous esophageal epithelium of a day 30 TCRβ3 mouse. Immunohistochemistry for CD3^+^ cells demonstrates markedly increased T cell numbers compared with a healthy control (CTRL) OTIIβ retrogenic mouse. (**G**) T cells from TCRβ7 retrogenic mice were isolated and stimulated as indicated. Proliferative response was measured at 72 h by ^3^H-thymidine incorporation. Circles indicate means of triplicates from individual mice. ****p ≤ 0.0001.

Of the additional group 1 TCRβ, none developed spontaneous EAE (Supplementary Fig. S2), though 2 of the 5 mice showed autoimmune features. Unprimed T cells from TCRβ4 mice proliferated strongly in response to MOG_35-55_ as measured both by ^3^H-thymidine incorporation and membrane-associated dye dilution assays (Fig. 4A–C). Therefore, like TCRβ1, TCRβ4 endows a large proportion of TCRαβ with specificity for the MOG_35-55_ autoantigen. TCRβ3T cells did not respond to MOG_35-55_ (Supplementary Fig. S3). However, with early engraftment these mice developed spontaneous alopecia and esophagitis (Fig. 4D–F). This was associated with prominent T cell infiltrates in these locations indicating that this CNS-associated public TCRβ can provoke alternative types of spontaneous autoimmunity.

There was no histologic or clinical evidence of disease in mice expressing any of the 4 group 2 TCRβ that were identified in a single CNS but public in the spleen (Supplementary Fig. S2). However, T cells from one of these, TCRβ7, proliferated weakly to MOG_35-55_. This was detectable by ^3^H-thymidine incorporation but not the less sensitive dye dilution assay (Fig. 4G). Mice expressing the 5 private group 3 TCRβ did not show evidence of spontaneous myelin reactivity or clinical or histologic disease (Supplementary Figs S2 and S3). In total, three public TCRβ, two in group 1 (TCRβ1, TCRβ4) and one in group 2 (TCRβ7), endowed unprimed T cells with MOG_35-55_ responsiveness in combination with endogenous TCRα. Two group 1 public TCRβ chains provoked spontaneous autoreactivity (TCRβ1, TCRβ3). No autoimmune phenotype was observed with the enforced expression of private (group 3) TCRβ.

## Discussion

We have previously shown that public TCRβ are preferentially incorporated into the CNS- infiltrating repertoire during MOG_35-55_ -induced EAE^17^. By assessing 15 distinct TCRβ *in vivo*, we further define the differential impact of public and private receptor chains implicated in the autoimmune response. Three public, but no private TCRβ were able to confer overt MOG_35-55_ - reactivity to unprimed T cells expressing endogenously rearranged TCRα. Enforced expression of two of six CNS-shared TCRβ provoked spontaneous autoimmunity in a mouse strain that does not otherwise develop spontaneous disease. This implies that public TCRβ can distort repertoire responses and foster reactivity to specific autoantigens.

One hypothesis for the preferential incorporation of public sequences into the autoimmune repertoire is that these sequences predispose TCRαβ toward self reactivity. Other repertoire studies have also identified public sequences among autoreactive T cells^11^,^25^,^26^. That public TCR may generically confer responsiveness to self-antigens is also suggested by our finding that transgenic expression of the public, EAE-associated TCRβ3 chain led to the development of spontaneous alopecia areata and not EAE. Therefore, a single TCRβ may promote reactivity to disease-associated autoantigens from different tissues. In this regard, it is noteworthy that a previously isolated TRBV13-2^+^ TCRβ from a MOG_35-55_ -specific hybridoma, 1MOG244.2, possesses two TCRα chains. Transgenic expression of one TCRαβ led to MOG_35-55_ reactive T cells. The second TCRαβ also provoked spontaneous alopecia areata, potentially suggesting a broader association between CNS and skin reactivities^27^. It is also possible that the preferential deployment of public TCR during EAE reflects a generic increase in TCR responsiveness to antigen. Indeed, though speculative, it is possible that TCR co-evolved with MHC such that increased recognition fitness is present in the high frequency public sequences that are most likely to form. Either model is supported by our finding that TCRs utilizing the public beta chain, TCRβ1, exhibit markedly enhanced sensitivity and maximal response when compared with control private TCR. Thus this public β chain may promote MOG_35-55_ recognition by endowing TCR with a particularly high functional avidity for antigen.

We found that 2 of the 6 group 1 TCRβ (CNS-shared and public), and altogether 3 public TCRβ broadly imposed MOG-specificity on TCRαβ. MOG-responsiveness was particularly prominent in mice expressing TCRβ1, where nearly one-third the number of CD4^+^ T cells from disease-free animals responding to αCD3 proliferated in response to MOG_35-55._ Unlike antibody-antigen interactions, which may rely on a single Ig chain, the TCR-MHC interface extensively involves both the TCRα and β surfaces. Implicitly, TCRβ1 dominates interactions defining specificity during MOG_35-55_ -IA^b^ recognition, and this is accompanied by more generic interactions with TCRα that are simply non-disruptive and provide requisite supplemental association energy for effective T cell stimulation. It cannot be excluded that TCRβ1 and other public TCRβ chains bind autoantigens in non-conventional manners that minimize reliance on the TCRα, and structural studies will be necessary to better resolve the physical nature of the reactivity imposed by these sequences^28^. In summary, we show that individual TCRβ sequences foster myelin antigen recognition in unprimed T cells. In a limited *in vivo* sampling of 15 transgenic TCRβ chains, this property was selectively observed in public TCR, providing a potential explanation for the preferential incorporation of public receptors into the autoimmune response.

## Methods

### Mice

C57BL/6 J (B6), B6.129P2-*Tcrb*^*tm1Mom*^/J (TCRβ^−/−^), B6.SJL-*Ptprc*^*a*^ *Pep3*^*b*^/BoyJ (CD45.1) and B6.129P2-*Rag1*^*tm1Mon*^/J (Rag1^−/−^) mice were purchased from The Jackson Laboratory (Bar Harbor, ME). Foxp3-GFP mice on a B6 background were obtained from Dr. A. Rudensky (NYU)^29^. Mice were bred under specific-pathogen-free conditions, and all animal experiments were conducted according to the experimental procedures approved by the Institutional Animal Care and Use Committee of St. Jude Children’s Research Hospital. Animal care was provided in Association for Assessment and Accreditation of Laboratory Animal Care (AAALAC) accredited animal barrier facilities at St. Jude Children’s Research Hospital.

### Flow cytometry

Cells were stained for 20 min at 4 °C in PBS containing 0.1% sodium azide and 2% (vol/vol) fetal bovine serum (FBS). Monoclonal antibodies specific for CD4 (clone RM4-5), CD8 (clone 53-6.7), TCRβ (clone H57-597), CD69 (clone H1-2F3), CD45.1 (clone A20) and CD45.2 (clone 104) were purchased from BD Biosciences. Intracellular staining of Foxp3 (clone FJK-16s) was performed using the Foxp3 Staining Buffer Set (eBioscience). For cytokine staining, cells were cultured for 4 h at 37 °C with Cell Stimulation Cocktail (eBioscience) in the presence of 10 μg/mL monensin (eBioscience), followed by fixation, permeabilization, and staining for IL-17A (clone eBio17B7, eBioscience) and IFN-γ (clone XMG1.2, BD Biosciences). Flow cytometric analysis was performed on an LSRFortessa (BD Biosciences) and analyzed with FlowJo software (Tree Star).

### Molecular subcloning

The TCRβ1 CDR3-Jβ segment was generated by annealing a pair of complementary oligonucleotides synthesized by St. Jude Hartwell Center (5′-TCGAGTTGGCTACCCCCTCTCAGACATCAGTGTACTTCTGTGCCAGCGGTGAGACTGGGGGAAACTATGCTGAGCAGTTCTTCGGACCAGGGACACGACTCACCGTCCTAGAA-3′; 5′-GATCTTCTAGGACGGTGAGTCGTGTCCCTGGTCCGAAGAACTGCTCAGCATAGTTTCCCCCAGTCTCACCGCTGGCACAGAAGTACACTGATGTCTGAGAGGGGGTAGCCAAC-3′). This was subcloned as a XhoI/BglII fragment into the previously cloned V-C region of the 1MOG244.2 TRBV13-2 TCRβ to synthetically recreate TCRβ1^23^. Other TCRβ constructs were similarly constructed. The OTII TCRβ was PCR amplified (5′-GCCGAATTCGCCACCATGTCTAACACTGCCTTC-3′; 5′-GTCACATTTCTCAGATCTTCTAG-3′) and then subcloned into the EcoRI/BglII sites of MSCV-TCRβ1-GFP to replace the TCRβ1V and J domains. For polycistronic TCRαβ constructs, TCRα and β chain cDNAs were linked as described using the *T*. *asigna* 2 A sequence and inserted in the MSCV-I-GFP retroviral vector, allowing stoichiometric production of each protein from a single message^30^,^31^.

### Generation of retrogenic mice

Retrogenic mice were generated as previously described^23^,^30^. Briefly, bone marrow cells from TCRβ^−/−^ mice were harvested and cultured in complete Click’s medium (Invitrogen) supplemented with 20% FBS, 20 ng/ml mIL-3, 50 ng/ml hIL-6, and 50 ng/ml mSCF (Pepro Tech) for 48 h. Hematopoietic progenitor cells (HPCs) were cocultured for 48 h with irradiated (1200 rads) GP + E86 retrovirus producer cells in complete Click’s medium supplemented as above and with 6 μg/ml polybrene. HPCs were harvested and injected i.v. into sublethally irradiated (450 rad) Rag1^−/−^ recipients. Engraftment was analyzed in peripheral blood by flow cytometry on day 28 after HPC transplantation.

### Clinical evaluation

Cohorts of retrogenic mice were clinically monitored for ≥120 days. Mice were submitted for histopathologic examination either during peak disease or after 120 days if healthy. Full necropsy, including of CNS tissues, was performed on at least three mice for each cohort. Paraffin-embedded tissue samples were stained with hematoxylin and eosin (H&E) and, where appropriate, CD3. The severity of spontaneous EAE was scored by using the predetermined qualitative and semi-quantitative criteria: 0, lesions absent, 1; minimal to mild inconspicuous lesions; 2, conspicuous lesions; 3, prominent multifocal lesions; 4, marked coalescing lesions.

### Bone marrow chimeric mice

HPCs from CD45.1^−^CD45.2^+^ TCRβ^−/−^ mice were transduced with TCRβ1 retrovirus as described above. Retrogenic HPCs were harvested and diluted with CD45.1^+^CD45.2^−^ congenic B6 bone marrow cells, and subsequently injected into irradiated (450 rad) CD45.1^+^CD45.2^−^ Rag1^−/−^ mice. Engraftment was analyzed at day 28 post-transplantation. Disease incidence was monitored for at least 60 days.

### Cell proliferation assays

Splenic CD4^+^ T cells from retrogenic mice were purified using MACS separation columns and anti-CD4 Ab (L3T4) coated microbeads (Miltenyi Biotec), and co- cultured at 5 × 10^4^ per well in 96-well plates with 2 × 10^5^ irradiated (3500 rad) syngeneic splenic APCs with or without 100 μg/ml MOG_35-55_ peptide for 72 h. Cells were pulsed with 1 μCi ^3^H- thymidine (PerkinElmer), and harvested 16 h later. Mouse T-Activator CD3/CD28 Dynabeads (Invitrogen) were added where indicated at a 1:1 bead-to-cell ratio. Alternatively, cells were labeled with 5 μM CellTrace Violet (Invitrogen) prior to stimulation according to the manufacturer’s instruction. Cells were stained with surface markers and 7-AAD (BD Biosciences) and T cell proliferation was measured by dye dilution. Proliferation analysis was performed with Flowjo software.

### Cytokine analysis

Culture supernatants from primary T cells were collected at 48 h and analyzed for IL-2, IL-4, IL-10, IFN-γ, and IL-1α using the Milliplex MAP mouse cytokine/chemokine immunoassay kit (Millipore) on a Luminex (Bio-Rad) instrument. For hybridomas, supernatant was assessed at 24 h for IL-2 only. For intracellular cytokine staining, cells were cultured with Cell Stimulation Cocktail and 10 μg/ml monensin (eBioscience), for 4 h at 37 °C, followed by fixation, permeabilization, and intracellular staining for IL-17A and IFN–γ.

### TCRαβ response to MOG_35-55_

TCRβ1 cDNA was linked with the indicated TCRα separated by the T2A sequence in the MSCV-I-GFP vector, and the polycistronic construct transduced into TCRαβ^−^CD4^+^ 4G4 hybridoma cells^23^. TCRαβ^+^ cells were sorted and co-cultured with 3 × 10^5^ (3500 rad) irradiated syngeneic splenic APCs and the indicated stimuli for 24 h. Culture supernatant was analyzed for IL-2 by sandwich ELISA (BD PharMingen).

### 5′RACE

T cells were isolated from the CNS of TCRβ1 retrogenic mice with disease scores ≥3. RNA was isolated and 5′ RACE performed using the 5′/3′ RACE Kit, 2nd Generation (Roche) following the manufacturer’s instructions. Briefly, full strand cDNA was synthesized from mRNA using specific primer 1 (5′-GGAGTCAAAGTCGGTGAACAG-3′). The mRNA template was degraded, polyA was added to the 3′ end of the cDNA, and the tailed cDNA was PCR amplified using the oligo (dT) anchor primer (5′-GACCACGCGTATCGATGTCGACTTTTTTTTTTTTTTTTV-3′) and a nested specific primer 2 (5′-CCTGAGACCGAGGATCTTTTAAC-3′). A second PCR reaction was performed with the PCR anchor primer (5′-GACCACGCGTATCGATGTCGAC-3′) and a nested specific primer 3 (5′-CAGGTTCTGGGTTCTGGAT-3′). PCR products were cloned into the TOPO TA vector (Invitrogen), sequenced, and sequences identified using the IMGT database (http://www.imgt.org).

### Statistics

Means, SDs, and Kaplan Meier curves were calculated in Excel or PRISM. Plots demonstrate mean ± 1SD. Two-tailed student t-tests were applied to compare any two groups and ANOVA for three or more groups. For multiple comparisons, significance is shown only for indicated groups. A *p* ≤ 0.05 was considered statistically significant.

## Additional Information

**How to cite this article**: Zhao, Y. *et al.* Autoimmune susceptibility imposed by public TCRβ chains. *Sci. Rep.* **6**, 37543; doi: 10.1038/srep37543 (2016).

**Publisher’s note:** Springer Nature remains neutral with regard to jurisdictional claims in published maps and institutional affiliations.

## Supplementary Material

Supplementary Information

## Figures and Tables

**Table 1:** TCRβ retrogenic mice.

TCR Name	TRBJ	CDR3 sequence	CNS shared (Total, n = 12)	CNS shared (Foxp3^−^)	CNS shared (Foxp3^+^)	SPL Shared (Total, n = 9)	SPL shared (Foxp3^−^)	SPL shared (Foxp3^+^)
**Group 1**, **CNS-shared**, **public**								
β1	2–1	ASGETGGNYAEQF	12	12	12	9	9	9
β2	2–7	ASGDRYEQY	12	12	8	9	9	9
β3	2–7	ASGYEQY	11	8	9	9	9	9
β4	1–2	ASGETANSDYT	11	6	10	9	9	9
β5	2–7	ASGDAGGSYEQY	10	8	10	9	9	9
β6	2–7	ASGDGEQY	9	4	9	9	9	9
**Group 2**, **CNS non-shared**, **public**								
β7	2–1	ASGEQQGTEQF	1	1 (22.6%)	1 (2.5%)	3	3	1
β8	2–7	ASGDGLGGSYEQY	1	1 (11.7%)	0	9	9	5
β9	1–6	ASGDVRGYNSPLY	1	0	1 (4.4%)	2	1	1
β10	1–2	ASGDGTSNSDYT	1	0	1 (3.8%)	9	9	2
**Group 3**, **non-shared**, **private**								
β11	2–5	ASGIGDTQY	1	0	1 (7.3%)	1	1	1
β12	2–7	ASGDAGTGYEQYF						
β13	2–4	ASGDWGGEDTLYF						
β14	2–4	ASGDETGGAYEQYF						
β15	2–3	ASGGGLGGTSAETLYF						
**Antigen non-specific negative control**								
OTIIβ	2–4	ASSLGGESQNTLYF						

TRBV13-2^+^ TCRβ chains that were shared in the indicated number of total CD4^+^, CD4^+^Foxp3^−^, and CD4^+^Foxp3^+^ populations in the CNS’ and spleens of mice with EAE were transduced into TCRβ^−/−^ HPCs to generate retrogenic mice. Sequences β1-β6 were identified in the CNS’ and spleens of multiple mice (CNS shared, public). Sequences β7-β10 were identified in the spleens of multiple mice, but in CNS tissue of only a single mouse (CNS non-shared, public). Sequence β11 was identified in the spleen and CNS of a single mouse, and sequences β12-β15 were isolated from TRBV13-2^+^ MOG_35-55_ -specific T cell hybridomas, and were not observed in any of the mice evaluated for the repertoire analyses (private). For TCRβ chains identified in a single mouse, the percent of total TRBV13-2^+^ TCR sequences in the CNS bearing the indicated sequence is listed in parentheses. OTIIβ comprises the TRBV13-2^+^ TCRβ chain from the OTII ovalbumin 323–229-specific TCR, and was assessed as a negative control.
