# Supplementary material for: Autoimmune susceptibility imposed by public TCRβ chains
Source: Sci Rep. 2016 Nov 21;6:37543. doi: 10.1038/srep37543 (PMC5116635; doi:10.1038/srep37543)
Supplement: Supplementary Information [file srep37543-s1.pdf]

Supplementary Information

Autoimmune susceptibility imposed by public TCRβ chains

Yunqian Zhao[a,1], Phuong Nguyen[a,1], Peter Vogel[a], Bofeng Li[a], Lindsay L. Jones[a], and Terrence L. Geiger[a,*]

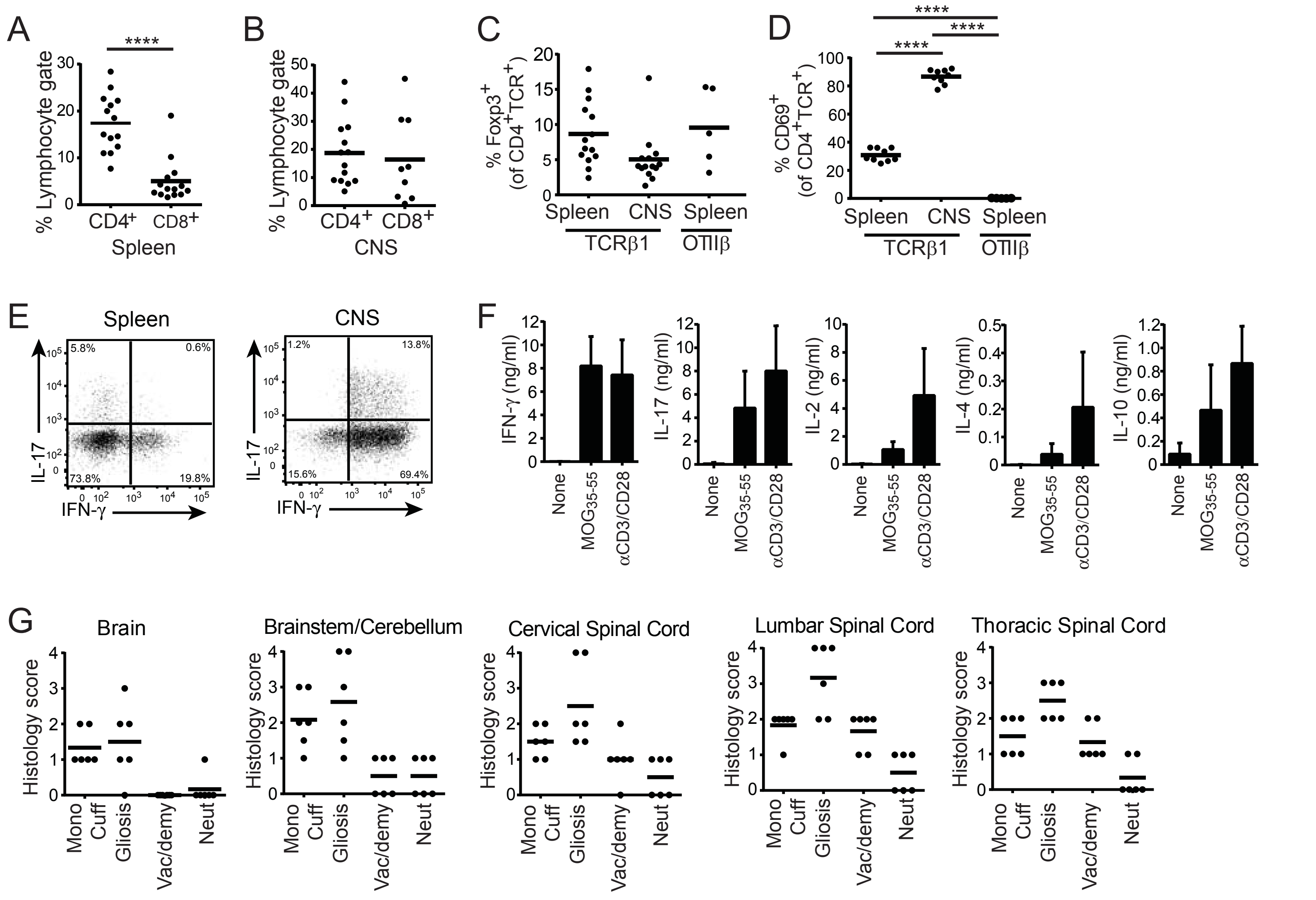

Supplementary Figure S1. Characterization of spontaneous EAE development in TCRβ1 retrogenic mice. Percent of CD4$^+$TCR$^+$ and CD8$^+$TCR$^+$ T cells within the lymphocyte gate in day 30-35 TCRβ mice in the spleen (A) and CNS (B). (C) Percent of CD4$^+$TCR$^+$ lymphocytes expressing GFP-Foxp3 in the spleens and CNS of TCRβ1 and control OTII TCRβ retrogenic mice is plotted. Groups are NS by ANOVA. (D) The percent of activated CD4$^+$TCR$^+$ cells was determined by CD69 activation marker expression in the spleen and CNS, and compared with results in the spleens of OTII TCRβ mice. (E) Splenic and CNS T cells from TCRβ1 mice with EAE were stimulated ex vivo with mitogen and IFNγ and IL-17 production measured by intracellular staining. Representative data is shown. Summary data is provided in Figure 1D. (F) Splenocytes from TCRβ1 mice with EAE were stimulated with MOG_{35-55} and secretion of the indicated cytokines measured by bead array. (G) Major organs from TCRβ1 mice with EAE were analyzed histologically. Inflammation was localized to the CNS. Scoring of the indicated CNS regions was performed by a blinded reviewer as described under Methods. ****, $p<0.0001$.

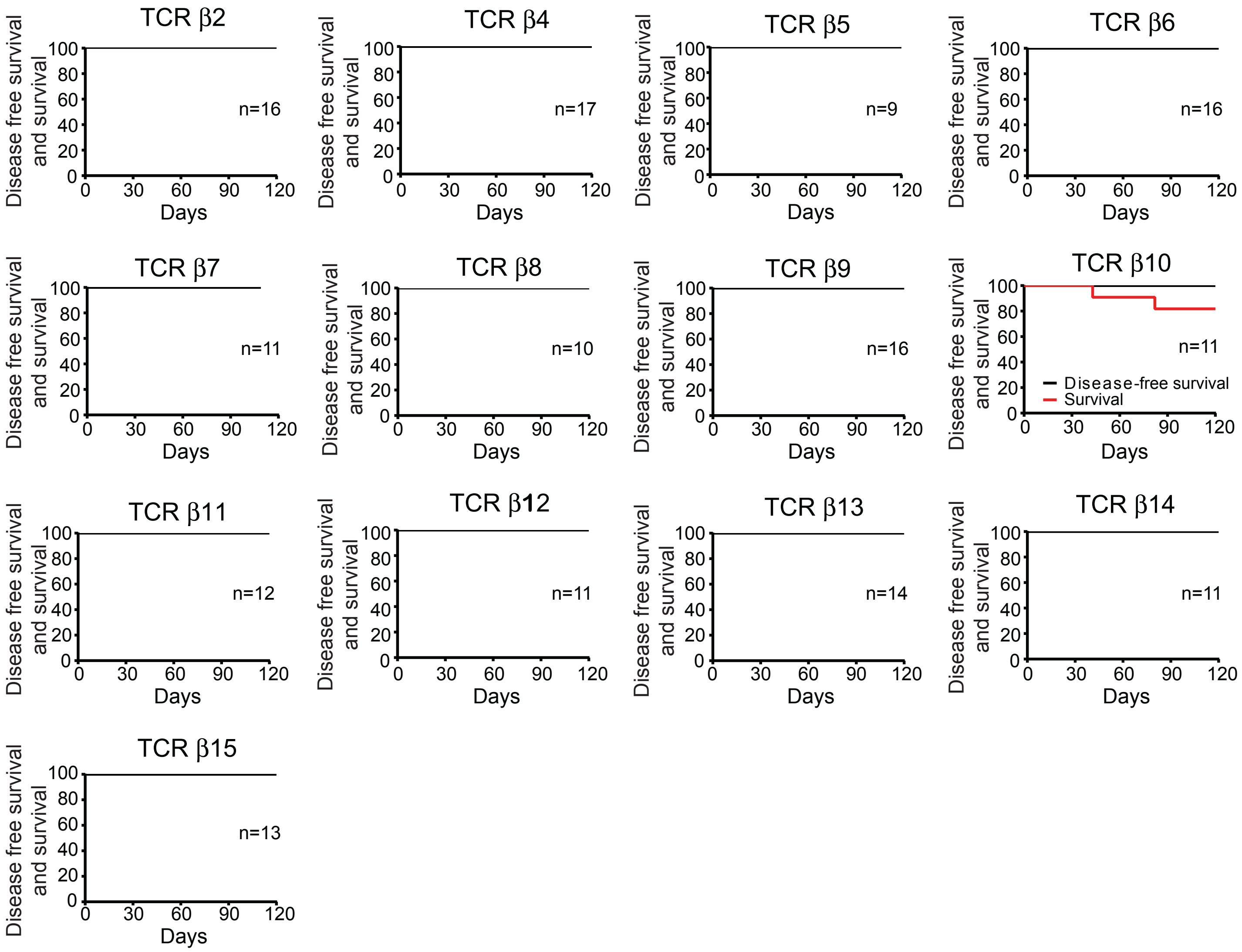

Supplementary Figure S2. Disease-free and overall survival of retrogenic mice. Kaplan Meier analysis of disease free and overall survival of group 1 (TCRβ1-6), group 2 (TCRβ7-10), and group 3 (TCRβ11-15) mice not otherwise shown in other figures. Disease free and overall survival was 100% over the 120 day observation period for all mice plotted except TCRβ10, of which two mice died without overt preceding illness or apparent cause.

Supplementary Figure S3

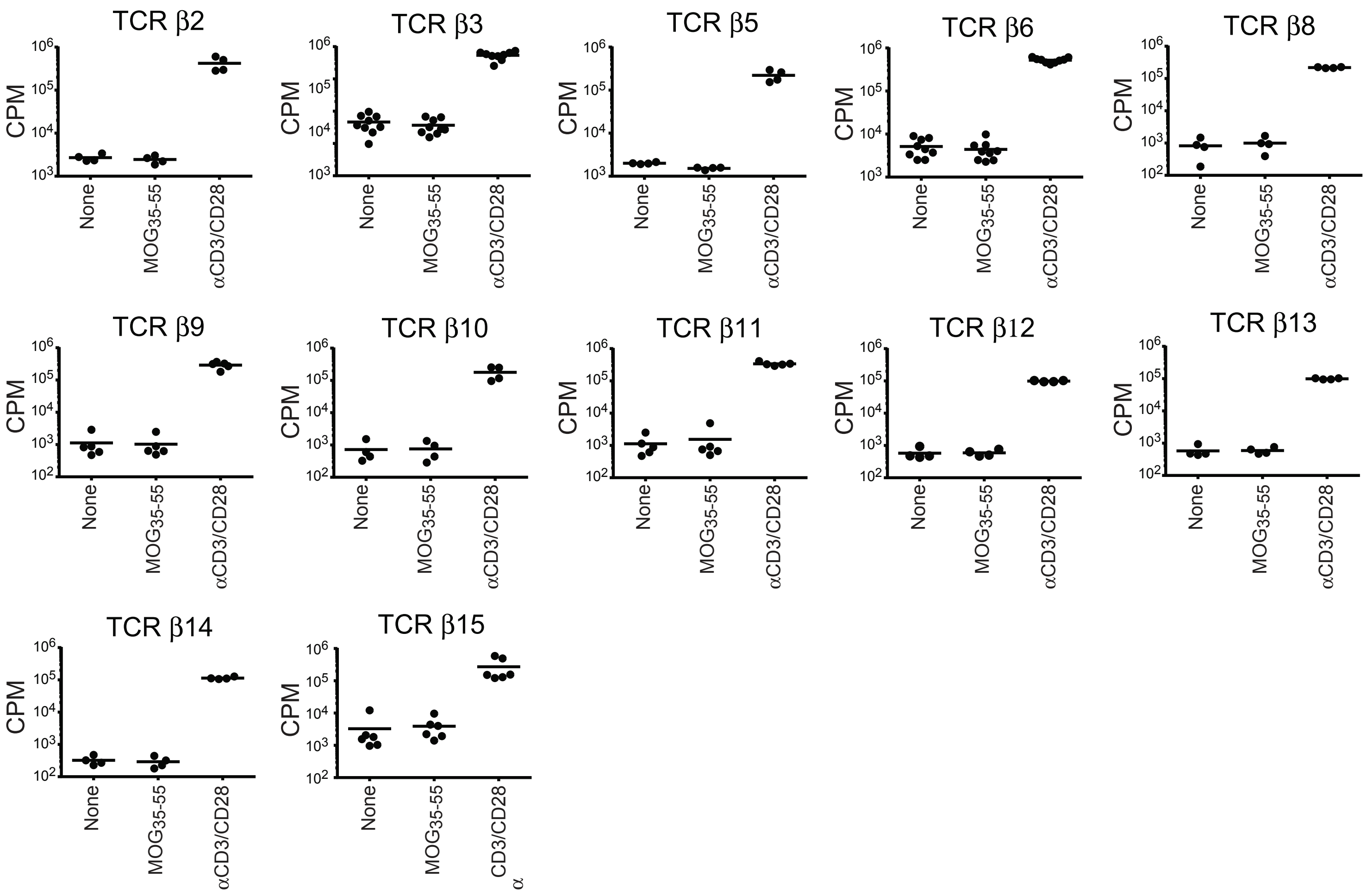

Supplementary Figure S3. MOG35-55 response of retrogenic mice. CD4+ T cells were purified from splees of the indicated disease-free retrogenic mice and cultured for 3 days in the presence of syngeneic irradiated splenic APCs in the absence of additional stimulation or with 100 μg/ml MOG35-55 or αCD3/CD28. Cultures were pulsed with 3H-thymidine at 72 hr and 3H incorporation measured. Circles indicate means of triplicates from individual mice. No significant differences were identified between unstimulated and MOG35-55 stimulated samples for the mice shown.

Mouse	Source	Cell Type	TRAV	TRAJ	CDR3
**5'RACE clones:**					
1	CNS	Foxp3-	TRAV13-2	TRAJ26	CAPPAHAQGLTF
1	CNS	Foxp3-	TRAV13D-1	TRAJ37	CALITGNTGKLIF
1	CNS	Foxp3-	TRAV14-2	TRAJ26	CAARTYAQGLTF
1	CNS	Foxp3-	TRAV14D-3	TRAJ31	CAARKNSNNRIFF
1	CNS	Foxp3-	TRAV4D-3	TRAJ49	CAAVTGYQNFYF
1	CNS	Foxp3-	TRAV4D-4	TRAJ42	CAASGGSNAKLTF
1	CNS	Foxp3-	TRAV4D-4	TRAJ57	CAAGQGGSAKLIF
1	CNS	Foxp3-	TRAV4N-3	TRAJ27	CAAGGYTGKLTF
1	CNS	Foxp3-	TRAV6D-6	TRAJ57	CALGDRGSAKLIF
1	CNS	Foxp3-	TRAV7-1	TRAJ21	CAVRKRSNYNVLYF
1	CNS	Foxp3-	TRAV7-2	TRAJ23	CAASMDYNQGKLIF
1	CNS	Foxp3-	TRAV7-3	TRAJ34	CAVSPQSSNTNKVVF
1	CNS	Foxp3-	TRAV7-4	TRAJ12	CAASGRTGGYKVVF
1	CNS	Foxp3-	TRAV7-4	TRAJ21	CAASARSNYNVLYF
1	CNS	Foxp3-	TRAV7-6	TRAJ33	CAASNYQLIW
1	CNS	Foxp3-	TRAV8-1	TRAJ39	CATPYNNAGAKLTF
1	CNS	Foxp3-	TRAV9N-2	TRAJ35	CVLSSGFASALTF
1	CNS	Foxp3-	TRAV9N-2	TRAJ35	CVLSAGFASALTF
1	CNS	Foxp3-	TRAV9N-3	TRAJ39	CAVSAINAGAKLTF
2	CNS	Foxp3-	TRAV12-2	TRAJ33	CALSARVNYQLIW
2	CNS	Foxp3-	TRAV14D-1	TRAJ57	CAASPQNQGGSAKLIF
2	CNS	Foxp3-	TRAV6-2	TRAJ17	CVLGDRRSAGNKLTF
2	CNS	Foxp3-	TRAV6-3	TRAJ45	CAMSGADRLTF
2	CNS	Foxp3-	TRAV7-4	TRAJ9	CAAGISNMGYKLTF
2	CNS	Foxp3-	TRAV7-4	TRAJ9	CAARISNMGYKLTF
3	CNS	Foxp3-	TRAV19	TRAJ24	CAVPASLGKLQF
3	CNS	Foxp3-	TRAV4D-3	TRAJ21	CAAGGYNVLYF
3	CNS	Foxp3-	TRAV7-3	TRAJ27	CAANTGKLTF
3	CNS	Foxp3-	TRAV7D-3	TRAJ37	CAVGGNTGKLIF
1	CNS	Foxp3+	TRAV13D-1	TRAJ39	CALVMNNNAGAKLF
1	CNS	Foxp3+	TRAV4D-3	TRAJ50	CAARSSSSFSKLVF
1	CNS	Foxp3+	TRAV4D-3	TRAJ42	CAAGGSNAKLTF
1	CNS	Foxp3+	TRAV4D-4	TRAJ57	CAAALNQGGSAKLIF
1	CNS	Foxp3+	TRAV4D-4	TRAJ57	CAAGQGGSAKLIF
1	CNS	Foxp3+	TRAV4N-3	TRAJ47	CAAVPMDYANKMIF
1	CNS	Foxp3+	TRAV7-2	TRAJ9	CAASWMGYKLTF
1	CNS	Foxp3+	TRAV7-3	TRAJ22	CAVSASSGSWQLIF
1	CNS	Foxp3+	TRAV7-3	TRAJ22	CAVSMSFGSWQLIF
1	CNS	Foxp3+	TRAV7-4	TRAJ12	CAASGRTGGYKVVF
1	CNS	Foxp3+	TRAV7D-3	TRAJ22	CAVSISGSWQLIF
2	CNS	Foxp3+	TRAV12-3	TRAJ39	CALRRGNAGAKLTF
2	CNS	Foxp3+	TRAV7-6	TRAJ32	CAVLGSSGNKLIF
2	CNS	Foxp3+	TRAV9D-3	TRAJ32	CALSPYGSSGNKLIF
2	CNS	Foxp3+	TRAV9D-3	TRAJ32	CALSPYESSGNKLIF
3	CNS	Foxp3+	TRAV21	TRAJ49	CILKTGYQNFYF
3	CNS	Foxp3+	TRAV6-5	TRAJ49	CILKTGYQNFYF
**Additional TRAV4 PCR clones:**					
3	CNS	Foxp3+	TRAV4D-3	TRAJ33	CAAPDSNYQLIW
3	CNS	Foxp3+	TRAV4D-3	TRAJ57	CAARQGGSAELIF
3	CNS	Foxp3+	TRAV4D-3	TRAJ40	CAAPGNYKYVF
3	CNS	Foxp3+	TRAV4D-3	TRAJ39	CAAGGDNAGAKLTF
3	CNS	Foxp3+	TRAV4D-3	TRAJ50	CAAIASSSFSKLVF
3	CNS	Foxp3+	TRAV4D-4	TRAJ57	CAAENQGGSAKLIF

Supplementary Table S1. Distinct TCRα chains isolated from CNS CD4$^+$ GFP-Foxp3$^+$ and GFP-Foxp3$^-$ T cells from TCRβ1 mice with EAE. TCRα were isolated by 5'RACE except for a subset of Foxp3$^+$ clones from mouse 3 that were isolated with TRAV4 and TCR Cα-specific primers.
